# Fibroepithelioma of Pinkus – confocal microscopy as a diagnostic tool^☆^

**DOI:** 10.1016/j.abd.2022.02.011

**Published:** 2023-06-29

**Authors:** Gabriella Campos-do-Carmo, Júlia Bozetti Lóss, Gustavo Costa Verardino

**Affiliations:** a Department of Dermatology, Gávea Medical Center, Rio de Janeiro, RJ, Brazil; b Department of Pathology, Microimagem, Rio de Janeiro, RJ, Brazil

Dear Editor,

A 36-year-old female patient presented with an asymptomatic pigmented lesion on her abdomen during total body mapping examination. The patient had no personal history of skin cancer, although she had already had atypical nevi removed.

Clinically, the lesion appeared as a brownish, sessile papule, measuring approximately 0.8 cm (Fig. 1). On dermoscopy, the presence of multiple bluish-gray dots were seen in the middle of the brownish amorphous area, as well as discrete thin vessels (Fig. 2).

**Figure 1:**
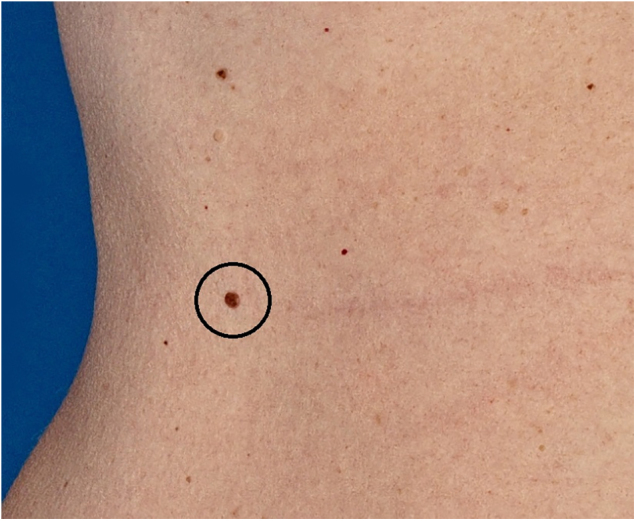
Brownish, sessile papule, measuring approximately 0.8 cm

**Figure 2:**
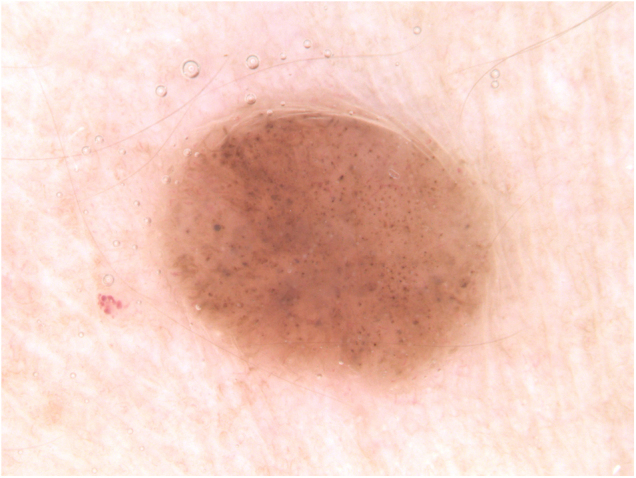
Dermoscopic image: presence of multiple bluish-gray dots in the middle of the brownish amorphous area and discrete thin vessels

Confocal reflectance microscopy was performed, an *in vivo* non-invasive imaging examination at the cellular level, which showed a fenestrated pattern, with the presence of refractive tumor cords, forming anastomoses and islets of basaloid cells, surrounded by fibrous, hyporefractive stroma. Basaloid cells in palisade arrangement were also observed at the periphery of the cords (Figure 3, Figure 4).

**Figure 3:**
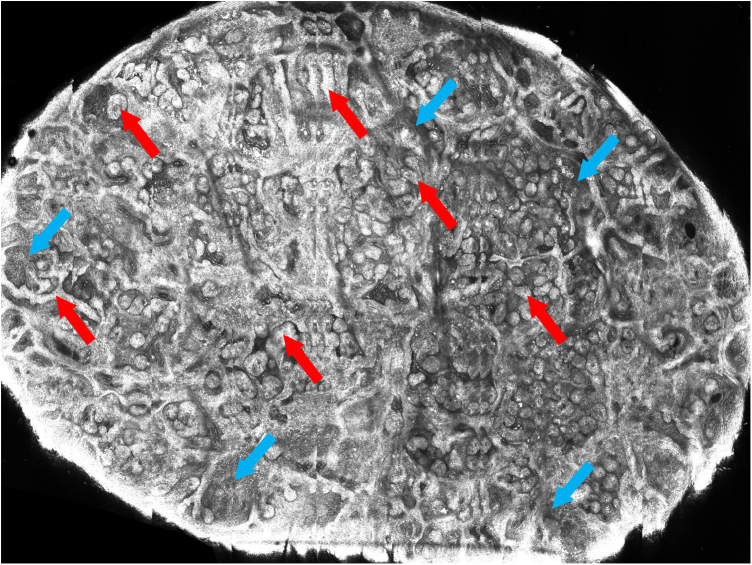
*In vivo* confocal microscopy mosaic: presence of interconnected cords and islets of tumor cells (red arrows), forming anastomoses, surrounded by fibrous stroma (blue arrows)

**Figure 4:**
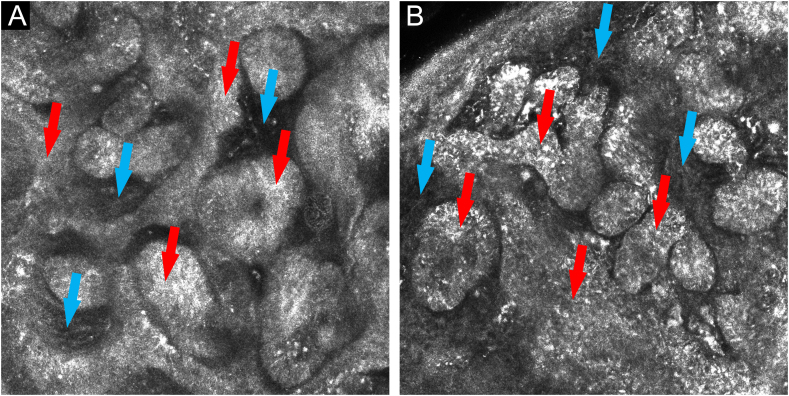
(A and B) *In vivo* confocal microscopy: islets and cords of tumor cells with a palisade periphery (red arrows), forming anastomoses, surrounded by fibrous stroma (blue arrows)

Histopathological analysis was performed and confirmed the diagnosis of fibroepithelioma of Pinkus (FeP), characterized by interconnected cords of tumor cells, with peripheral palisade, surrounded by fibrous stroma (Fig. 5A). Immunohistochemical analysis showed the expression of the Ber-EP4 in the cords of tumor cells (Fig. 5B). At the lower limit, structures called germ-papillae, characteristic of FeP, were observed. The histological arrangement corresponds to the fenestrated pattern on confocal microscopy, allowing the safe diagnosis of FeP by this technique.

**Figure 5:**
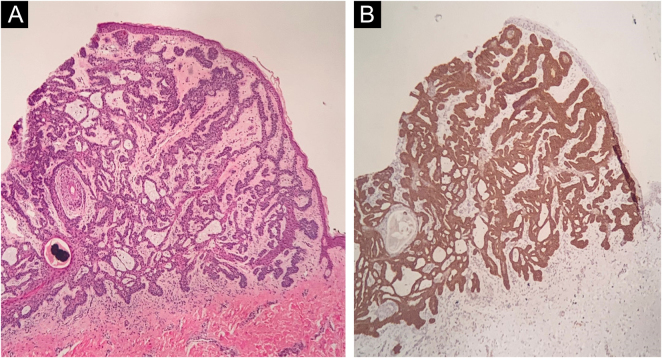
(A) Histopathology of the lesion showing cords of tumor cells with a palisaded periphery (Hematoxylin & eosin, ×40). (B) Immunohistochemistry demonstrating the expression of BER-EP4 in the cords of tumor cells (×40)

FeP is a peculiar and uncommon subtype of basal cell carcinoma, which can be clinically similar to benign tumor lesions, such as intradermal nevus, fibroepithelial polyp, and seborrheic keratosis, among others, which are not routinely excised. Classically, FeP presents as a solitary, cupuliform, normochromic, or brownish papule. Dermoscopy may show polymorphous vessels (thin, focused, short arboriform, dotted), short white lines, milia-like cysts, brownish-gray areas, and bluish-gray dots.^1^, ^2^, ^3^

Histopathology is considered essential for diagnosis. It was described by Pinkus as peculiar and unmistakable,^4^ it features filaments or anastomosing cords of basaloid cells projecting downwards from the epidermis in a fenestrated pattern, surrounded by abundant fibrous stroma. The periphery of the cords is formed by columnar cells, in palisade arrangement.^5^, ^6^ Immunohistochemistry using the Ber-EP4 marker is a useful tool to diagnose neoplasms with follicular germinative differentiation and can be used to corroborate the histopathological diagnosis.^7^

*In vivo* confocal microscopy also shows the characteristic fenestrated pattern. At the level of the dermal-epidermal junction, hyporefrective spaces can be observed, which correspond to the fibrous stroma, surrounded by cords of tumor cells, with greater refraction. Cords and islets of tumor cells show a palisade arrangement at the periphery and canalicular vessels may also be observed.^2^, ^3^

FeP often goes underdiagnosed on clinical examination. The clinical characteristics are often nonspecific and dermoscopy may not be sufficient to confirm or rule out other hypotheses. However, the specific pattern revealed by confocal microscopy corresponds to the peculiar histopathological characteristics of FeP, making its diagnosis possible with a higher level of safety and specificity.^5^, ^8^

## Financial support

None declared.

## Authors' contributions

Gabriella Campos do Carmo das Chagas: Design and planning of the study; data collection, or analysis and interpretation of the data; drafting and editing of the manuscript or critical review of important intellectual content; collection, analysis and interpretation of data; effective participation in research orientation; intellectual participation in the propaedeutic and/or therapeutic conduct of the studied cases; critical review of the literature; approval of the final version of the manuscript.

Júlia Bozetti Lóss: Data collection, or analysis and interpretation of data; drafting and editing of the manuscript or critical review of important intellectual content; collection, analysis and interpretation of data; intellectual participation in the propaedeutic and/or therapeutic conduct of the studied cases; critical review of the literature; approval of the final version of the manuscript.

Gustavo Costa Verardino: Data collection, or data analysis and interpretation; drafting and editing of the manuscript or critical review of important intellectual content; collection, analysis and interpretation of data; approval of the final version of the manuscript.

## Conflicts of interest

None declared.
